# Correlation of Stress and Oral Inflammatory Burden in Patients With Chronic Periodontitis in a Sample Population From Bhopal: A Randomized Clinical Study

**DOI:** 10.7759/cureus.70974

**Published:** 2024-10-07

**Authors:** Anuj Singh Parihar, Sumit Narang

**Affiliations:** 1 Periodontics, People’s Dental Academy, Bhopal, IND

**Keywords:** chronic periodontitis, oral inflammatory burden, pisa (periodontal inflamed surface area), randomized clinical study, stress

## Abstract

Introduction

Chronic periodontitis is a prevalent inflammatory disease that leads to the destruction of tooth-supporting structures. Psychological stress is a potential risk factor for periodontitis, potentially exacerbating inflammation and impairing treatment outcomes. This study aims to explore the correlation between chronic stress and oral inflammatory burden, as measured by the Periodontal Inflamed Surface Area (PISA), in a sample population from Bhopal, India.

Methods

This randomized clinical study included 1,250 participants, divided into three groups: Group A (control, n=250), Group B (chronic periodontitis, n=500), and Group C (post-treatment chronic periodontitis, n=500). Participants underwent a comprehensive periodontal examination, including the calculation of PISA, and completed the Perceived Stress Scale-10 (PSS-10) to assess stress levels. Statistical analysis included Pearson’s correlation to assess the relationship between PSS-10 scores and PISA, with comparisons among groups using analysis of variance or Kruskal-Wallis tests.

Results

Group B exhibited significantly higher periodontal parameters and PSS-10 scores than Group A and Group C (p<0.001 for all comparisons). Group C showed significant improvements in both periodontal parameters and PSS-10 scores following treatment (p<0.001). A positive correlation was observed between PSS-10 scores and PISA in Group B (r=0.62, p<0.001), indicating that higher perceived stress was associated with increased oral inflammation in untreated chronic periodontitis. This correlation persisted after adjusting for confounders, including age, sex, and socioeconomic status.

Conclusions

Chronic stress is significantly associated with increased oral inflammatory burden in patients with chronic periodontitis, suggesting that stress may act as an independent risk factor for disease progression. Periodontal therapy reduces oral inflammation and alleviates psychological distress. Integrating stress management into periodontal treatment plans may enhance patient outcomes, highlighting the importance of a holistic approach to periodontal care.

## Introduction

Chronic periodontitis is a common inflammatory disease that progressively destroys tooth-supporting structures, significantly impacting global oral health [1,2]. Periodontitis, primarily triggered by bacterial infection in dental plaque, results in the breakdown of periodontal tissues, including the gums, periodontal ligament, and alveolar bone [3].

While risk factors such as poor oral hygiene and smoking are well-established, the role of psychological stress in periodontitis has gained recent attention [4]. Chronic stress may affect periodontal disease by modulating host immune responses through neuroendocrine and immune pathways [5]. Studies suggest that individuals experiencing chronic stress have a higher prevalence of periodontitis, with stress potentially exacerbating inflammation, impairing wound healing, and reducing the efficacy of periodontal treatment [6]. Additionally, periodontitis itself may contribute to chronic low-grade inflammation, amplifying the stress response [7]. The Periodontal Inflamed Surface Area (PISA), introduced by Nesse et al. in 2008 [8], quantifies the inflammatory burden in periodontitis, offering a useful tool for assessing disease activity and potential systemic effects [9]. This study aims to assess the correlation between chronic stress and oral inflammatory burden, as measured by PISA, in a sample population from Bhopal, India.

## Materials and methods

This randomized clinical study was conducted at the Outpatient Department of Periodontics at People’s Dental Academy in Bhopal, Madhya Pradesh, India. The study design was approved by the University Doctoral Committee of People’s University, Bhopal (01 PU/DAA/Ph.D./Registration/11/707C), and the clinical trial was registered with Clinical Trials Registry - India (CTRI/2023/09/056213). Written informed consent was obtained from all participants. Participants were selected using consecutive sampling from the Outpatient Department. A total of 1,250 participants were recruited and divided into three groups: Group A (control, n=250), consisting of healthy individuals without periodontitis; Group B (n=500), comprising patients with chronic periodontitis; and Group C (n=500), consisting of patients who had received periodontal treatment at least six months prior.

Participants were 18 years or older. For Groups B and C, additional inclusion criteria included a diagnosis of chronic periodontitis with Probing Pocket Depths (PD) of >4 mm in more than 30% of sites. All participants were non-smokers. Exclusion criteria included current smoking, pregnancy or lactation, systemic diseases, such as diabetes or immunosuppressive conditions, and age over 60 years.

We collected demographic and medical history data using a standardized form. This included age, sex, and relevant medical and dental history. Socioeconomic status was assessed using a composite index incorporating the following: (i) Education level: Participants were categorized based on their highest level of education attained (e.g., no formal education, primary education, secondary education, higher education); (ii) Occupation: Participants' occupations were classified based on skill level and income potential (e.g., unskilled labor, skilled labor, professional); and (iii) Monthly household income: Participants provided information about their estimated monthly household income, which was then categorized into quartiles based on local population data.

These three factors were combined to assign each participant a socioeconomic status category (low, middle, or high). This composite index allowed us to account for multiple dimensions of socioeconomic status and its potential impact on oral health and stress levels. A calibrated periodontist performed comprehensive periodontal examinations, including assessments of the Oral Hygiene Index Simplified, Gingival Index, PD, Clinical Attachment Level, and PISA. PISA was calculated using the method described by Nesse et al. (2008) [8]. Perceived stress levels over the past month were assessed using the Perceived Stress Scale-10 (PSS-10), a validated and reliable tool for measuring subjective stress.

Statistical analysis

Data were analyzed using IBM SPSS Statistics for Windows, Version 23.0. (IBM Corp., Armonk, USA). Descriptive statistics summarized demographic and clinical characteristics. Pearson’s correlation coefficient assessed the relationship between chronic stress (PSS-10 scores) and oral inflammatory burden (PISA). Group comparisons of periodontal parameters and stress levels were performed using analysis of variance or Kruskal-Wallis tests as appropriate. A p-value <0.05 was considered statistically significant.

## Results

The study included 1,250 participants, with a mean age of 38.5 ± 12.3 years (standard deviation). Table 1 presents the demographic characteristics of the three study groups. There were no statistically significant differences between the groups in terms of age, sex distribution, or socioeconomic status (p>0.05 for all comparisons), ensuring comparability for the primary outcome analysis.

**Table 1:** Demographic characteristics of the study population SD, standard deviation

Characteristic		Group A (Control, n=250)	Group B (Pre-treatment, n=500)	Group C (Post-treatment, n=500)	p-value
Age in years, mean ± SD		37.2 ± 11.8	39.1 ± 12.6	38.8 ± 12.1	0.21
Sex, n	Male	120	245	250	0.87
	Female	130	255	250	
Socioeconomic status, n	Low	80	195	200	0.93
	Middle	120	210	205	
	High	50	95	95	

Table 2 summarizes the three groups’ clinical periodontal parameters and PSS-10 scores. Group B (pre-treatment chronic periodontitis) had significantly higher mean scores for all periodontal parameters and PSS-10 compared to Group A (control) and Group C (post-treatment; p<0.001 for all comparisons), indicating greater periodontal inflammation and higher perceived stress in patients with active chronic periodontitis. Group C demonstrated significant improvements in all periodontal parameters and PSS-10 scores compared to Group B (p<0.001 for all comparisons), suggesting the beneficial effects of periodontal therapy on both oral health and psychological well-being.

**Table 2:** Clinical periodontal parameters and stress levels of the study population OHI-S, Oral Hygiene Index Simplified; GI, Gingival Index; PD, Probing Pocket Depth; CAL, Clinical Attachment Level; PISA, Periodontal Inflamed Surface Area; PSS-10, Perceived Stress Scale-10; SD, standard deviation

Parameter	Group A (Control), mean ± SD	Group B (Pre-treatment), mean ± SD	Group C (Post-treatment), mean ± SD	p-value
OHI-S	0.8 ± 0.3	2.3 ± 0.6	1.2 ± 0.4	<0.001
GI	0.9 ± 0.4	2.1 ± 0.5	1.1 ± 0.3	<0.001
PD (mm)	2.1 ± 0.5	4.8 ± 1.2	2.5 ± 0.7	<0.001
CAL (mm)	1.2 ± 0.4	3.5 ± 1.0	1.6 ± 0.5	<0.001
PISA (cm^2^)	3.2 ± 1.8	12.5 ± 4.3	4.6 ± 2.1	<0.001
PSS-10	15.3 ± 4.2	22.7 ± 5.1	17.2 ± 4.5	<0.001

We noted a significant positive correlation between PSS-10 scores and PISA in Group B (r=0.62, p<0.001). This finding indicates that higher levels of perceived stress are associated with increased oral inflammatory burden in patients with active chronic periodontitis. This correlation remained significant after adjusting for potential confounders, including age, sex, and socioeconomic status, suggesting that stress is an independent predictor of oral inflammation in this population. No significant correlation was found between PSS-10 and PISA scores in Group A or Group C.

## Discussion

This randomized clinical study demonstrates a significant association between chronic stress and increased oral inflammatory burden in patients with chronic periodontitis. Higher levels of perceived stress, as measured by the PSS-10, were correlated with greater periodontal inflammation, reflected by elevated PISA scores. This relationship remained significant after adjusting for confounders, suggesting that stress is an independent risk factor for periodontal disease progression.

These findings align with existing literature emphasizing the interplay between the oral and systemic immune systems [10]. Chronic stress affects neuroendocrine and immune pathways, disrupting the balance between host defense mechanisms and microbial challenges in the oral cavity [11]. This disruption can increase susceptibility to periodontal pathogens, impair wound healing, and heighten inflammatory responses, contributing to tissue destruction and disease progression [12]. The significant reduction in both PISA scores and stress levels in post-treatment patients highlights the benefits of periodontal therapy in reducing oral inflammation and psychological distress [13]. This suggests a bidirectional relationship between stress and periodontal disease, where successful treatment of one may improve the other [14].

These findings highlight the importance of a holistic approach to periodontal care. In addition to traditional treatment, incorporating stress management strategies, such as mindfulness, relaxation exercises, or cognitive behavioral therapy, may enhance patient outcomes and improve quality of life [15]. This study also contributes to the growing recognition of the oral-systemic connection, particularly in chronic inflammatory diseases. Managing periodontal disease and addressing associated stress may help reduce the overall burden of chronic inflammation and its systemic consequences [16].

Limitations

This study has several limitations. First, its cross-sectional design limits the ability to establish causal relationships between stress and periodontal inflammation. Second, reliance on self-reported stress levels, measured by the PSS-10, may introduce recall bias and individual interpretation. Third, the study population was limited to patients attending a single dental institution in Bhopal, which may affect the generalizability of the findings.

Furthermore, while this study focused specifically on oral inflammatory burden as measured by PISA, we acknowledge that subclinical inflammatory processes elsewhere in the body could contribute to the overall inflammatory load and potentially influence the relationship between stress and periodontitis. Future research could expand upon our findings by incorporating measures of systemic inflammation, such as C-reactive protein or interleukin-6, to provide a more comprehensive understanding of these complex interactions.

## Conclusions

We conducted this study to assess the influence of chronic stress on periodontal disease progression, particularly its effect on oral inflammatory burden. Our results underscore the detrimental impact of chronic stress on periodontal health in individuals with chronic periodontitis. The positive correlation between perceived stress and oral inflammatory burden highlights the need for a comprehensive approach to periodontal care that addresses physical and psychological well-being. The improvements in periodontal parameters and stress levels following treatment suggest that managing oral inflammation can also positively affect psychological health. Integrating stress management into periodontal treatment plans may enhance patient outcomes and quality of life.
